# Drug repurposing of Nifuratel against methicillin-resistant *Staphylococcus aureus* through proton motive force disruption

**DOI:** 10.3389/fcimb.2025.1738031

**Published:** 2026-01-09

**Authors:** Pengfei She, Bingqin Qin, Kening Lin, Di Zhang

**Affiliations:** 1 Department of Laboratory Medicine, The Third Xiangya Hospital of Central South University, Changsha, Hunan, China; 2 Department of Laboratory Medicine, Chenzhou Third People’s Hospital, Chenzhou, Hunan, China

**Keywords:** methicillin-resistant, *Staphylococcus aureus*, drug repurposing, molecular dynamics, Nifuratel, proton motive force

## Abstract

**Introduction:**

The diminishing efficacy of conventional antibiotics against methicillin-resistant *Staphylococcus aureus* (MRSA) necessitates novel therapeutic strategies. Drug repurposing represents a promising approach. This study investigates the antibacterial potential of Nifuratel, a repurposed agent, against MRSA.

**Methods:**

*In vitro* antibacterial activity was assessed against type strains and clinical isolates via minimum inhibitory concentration (MIC) and minimum bactericidal concentration (MBC) assays. The propensity for resistance development was evaluated. Sub-MIC effects on key virulence phenotypes-biofilm formation, hemolysis, auto-aggregation, and surface spreading-were examined. The mechanism of action was investigated using transmission electron microscopy, fluorescence probes, and molecular dynamics simulations. Efficacy and biocompatibility were evaluated *in vivo* using murine abscess and wound infection models, with assessments of bacterial load, inflammation, wound healing, hemolysis, and organ toxicity.

**Results:**

Nifuratel exhibited potent bactericidal activity with MICs of 2–8 µg/mL and MBCs of 8–16 µg/mL, and a low propensity for resistance development. At sub-MIC concentrations, it significantly suppressed MRSA virulence phenotypes. Mechanistic studies revealed that Nifuratel disrupts the proton motive force by dissipating both the transmembrane potential and proton gradient, without causing direct membrane damage. *In vivo*, Nifuratel treatment significantly reduced bacterial loads, attenuated inflammation, and promoted wound healing comparably to fusidic acid. The compound demonstrated excellent biocompatibility with minimal hemolysis and no observed organ toxicity.

**Discussion:**

These results identify Nifuratel as a promising repurposed antimicrobial agent against MRSA. Its dual capability to exert direct bactericidal activity by disrupting PMF and attenuate key virulence factors, combined with a favorable resistance profile and biocompatibility *in vivo*, supports its potential for further therapeutic development.

## 1 Introduction

Methicillin-resistant *Staphylococcus aureus* (MRSA) represents a critical global health threat, causing an estimated 100,000 deaths worldwide in 2019 (Chen et al., 2022). The mortality rate of MRSA-related bacteremia is 20%, higher than drug-susceptible strains (Channabasappa et al., 2018b). The resistance of MRSA extends beyond β-lactams to fluoroquinolones, macrolides, and glycopeptides, severely limiting treatment options. Healthcare-associated infections prolong hospitalization and increase treatment costs compared to susceptible infections (Channabasappa et al., 2018a; Mohiuddin et al., 2022). In addition, *S. aureus* biofilms on medical devices such as catheters and prosthetics create physical barriers that reduce antibiotic penetration through extracellular polymeric substance (Yoshii et al., 2017). The biofilm formation ability by *S. aureus* largely increased its resistance to antibiotics in clinical settings (Worthington et al., 2012). Thus, these complications necessitate the development of novel antimicrobials.

Drug repurposing leverages established safety profiles to address antimicrobial resistance within shortened development timelines (Zheng et al., 2022). For example, auranofin, a small molecule used for the treatment of rheumatoid arthritis, inhibits bacterial thioredoxin reductase through gold ion coordination (Liu et al., 2022), while disulfiram, an Aldehyde Dehydrogenase-1 inhibitor, disrupts bacterial metal ion homeostasis via copper chelation (Thakare et al., 2019). The breast cancer drug toremifene was repurposed as an antibacterial agent against oral pathogens like *P. gingivalis* and *S. mutans* by disrupting microbial cell membranes (Gerits et al., 2017). These cases exemplify the transformative potential of repositioning existing pharmacotherapies. Meanwhile, drug repurposing is a great approach in combinational therapy. The adjunctive use of various repurposed natural products or non-antibiotic agents (such as statins and metformin) with antibiotics has been demonstrated to improve tuberculosis treatment outcomes and mitigate the adverse effects of the antibiotics (Khameneh et al., 2019). And Morley et al (Morley et al., 2020). reported that the FDA-approved drug cholestyramine could be repurposed as an “anti-antibiotic” to sequester daptomycin in the gut, aiming to preserve its systemic therapeutic effects while preventing the emergence of resistance in the microbiome. This adjunctive strategy reduced the fecal shedding of daptomycin-resistant Enterococcus by up to 80-fold, offering a promising proof-of-concept for curbing the spread of antibiotic resistance. In addition, nonsteroidal anti-inflammatory drugs inhibit toxin-regulating two-component systems by competitive interaction with the ATP-binding pocket of SaeR (Jiang et al., 2023), while retinoid derivatives block staphyloxanthin biosynthesis through crtM enzyme inhibition (Kim et al., 2018). Such anti-virulence adjunctives neutralize pathogenic weapons without affecting bacterial growth, which could preserve commensal microbiota while disabling key virulence determinants.

The proton motive force (PMF), comprising transmembrane electrical potential (Δψ) and proton concentration gradient (ΔpH), serves as the primary energy currency for bacterial viability (Seo et al., 2024). Disrupting this fundamental process circumvents existing resistance mechanisms while maintaining broad-spectrum activity against Gram-positive pathogens (Mohiuddin et al., 2022; Yang et al., 2023). Crucially, PMF disruption demonstrates selective toxicity against bacterial cells due to divergent energy conservation systems in mammalian mitochondria (Yang et al., 2023).

Originally approved for genitourinary infections, Nifuratel demonstrates STAT3 pathway inhibition with documented antitumor (Zheng et al., 2017) and antiallergic applications (Lee et al., 2023). Preliminary studies suggest antibacterial properties against Trichomonas and Candida (Gruneberg and Leakey, 1976), however, its activity against *S. aureus* remains unexplored. The present study evaluated the *in vitro* and *in vivo* antimicrobial effects of Nifuratel against MRSA and its virulence factors. And the underlying antimicrobial mechanisms of Nifuratel were further explored.

## 2 Materials and methods

### 2.1 Strains, chemicals and culture conditions

The type strains *S. aureus* ATCC 25923 (MSSA) and *S. aureus* ATCC 43300 (MRSA) were purchased from the American Type Culture Collection (ATCC). Clinical MRSA isolates, including strain SA-11, were obtained from the Third Xiangya Hospital of Central South University. All clinical strains were identified using the VITEK 2 Compact system (bioMérieux, France) and confirmed by Matrix-Assisted Laser Desorption/Ionization Time-of-Flight (MALDI-TOF) mass spectrometry (BD, Germany). Bacterial cultures were grown in Tryptic Soy Broth (TSB) (Solarbio, Beijing, China). Nifuratel, vancomycin (VAN), tetracycline (TCY), ciprofloxacin (CIP), and other antimicrobial agents were purchased from MedChem Express (New Jersey, USA) and dissolved in either deionized water or dimethyl sulfoxide (DMSO) as stock solutions for subsequent experiments.

### 2.2 Minimal inhibitory concentration and minimal bactericidal concentration determination by micro-broth dilution assay

The MICs and MBCs of *S. aureus* were tested in cation-adjusted Mueller-Hinton (MH) broth. Briefly, 50 μL of 2-fold serially diluted Nifuratel were added into a 96-well plate. Then, 50 μL of the bacterial suspensions were added to the plate to yield 5×10^5^ CFU/mL final inoculum. After incubated at 37°C for 24h, MICs were defined as the lowest concentration showing complete growth inhibition. Further, Minimum Bactericidal Concentration (MBC) is determined by subculturing broth from MIC wells showing no visible growth onto sheep blood agar plates after overnight incubation (CLSI, 2024).

### 2.3 Kirby-Bauer assay

*S. aureus* was adjusted to 0.5 McFarland (McF) standard in saline, and spread evenly onto MH agar plates by using a moist swab. Then, sterile empty disks in the presence or absence of Nifuratel were aseptically placed on the agar surface. The plates were incubated at 37°C for 16–18 h, and the inhibition zones were measured with a caliper (She et al., 2022).

### 2.4 Growth inhibition assay

The assay was conducted following the aforementioned micro-broth dilution method, with minor modifications. Briefly, Nifuratel was serially diluted in TSB or MH broth, and equal volumes of log-phased *S. aureus* cultures were added to a 96-well plate to the final density of ~5 × 10^5^ CFU/mL (100 µL/well). After 16h incubation at 37°C, bacterial growth was measured at 630 nm using a microplate reader.

### 2.5 Time-kill assay

Log-phased *S. aureus* was diluted and inoculated at ~1 × 10^6^ CFU/mL in TSB medium in the presence or absence of antimicrobial agents at indicated concentrations. The bacterial suspension was incubated at 37°C 180 rpm. Then, aliquots of the bacterial suspension were serially diluted in saline and plated on sheep blood agar at intervals of 0, 2, 4, 8, 12, and 24h, respectively. After incubation at 37°C for 24h, viable colonies were counted to calculate log_10_ CFU/mL reductions (Visca et al., 2019).

### 2.6 SYTO9/propidium iodide viability staining

Bacterial viability was assessed using dual-fluorescence staining with the LIVE/DEAD^®^ BacLight™ Bacterial Viability Kit (L7012, Thermo Fisher Scientific). Briefly, bacterial suspensions (∼1×10^6^ CFU/mL) or biofilms were treated with a premixed dye cocktail, including SYTO9 and PI, according to the manufacturer’s protocol. After incubated in the dark for 15 min at room temperature, the samples were washed in 1×phosphate-buffered saline (PBS) and immediately visualized under confocal laser scanning microscopy (CLSM) (LSM800, ZESS, Germany) with dual-channel detection.

### 2.7 Biofilm inhibition assay

Bacterial cultures were grown overnight in TSB supplemented with 0.5% glucose (TSB-G). The cultures were then diluted 1:100 in fresh medium, and aliquoted (100 μL/well) into a sterile 96-well plate. Then, equal volume of 2-fold diluted Nifuratel were added into each well. After 24 h of static incubation at 37°C, planktonic cells were removed by gentle washing with PBS. For crystal violet staining, biofilms were added with 0.15% crystal violet for 10 min, washed, and solubilized in 95% ethanol. Absorbance was measured at 570 nm to quantify total biofilm biomass (Xu et al., 2016). For the CFU counting assay, the supernatant in each well was serially diluted 10-fold with 1× PBS and spotted onto sheep blood agar. Then, the supernatant was removed, and the adherent biofilms were gently washed with 1× PBS. Subsequently, 150 µL of PBS was added to each well, and the biofilms were thoroughly dispersed and homogenized using pipette tips. After appropriate dilution, the suspensions were plated onto sheep blood agar. All the agars were incubated overnight at 37°C, after which CFUs were enumerated.

### 2.8 Biofilm eradication assay

Bacterial cultures were grown overnight in TSB supplemented with 0.5% glucose (TSB-G). The cultures were then diluted 1:100 in fresh medium, and aliquoted (200 μL/well) into a sterile 96-well plate. After 24 h of static incubation at 37°C, planktonic cells were removed by gentle washing with PBS, and the remined biofilms were treated with 2-fold diluted Nifuratel. After incubation for another 24h, the biofilms were washed again and quantified by crystal violet staining (Nair et al., 2016; Xu et al., 2016) and CFU counting assay, respectively, as described above.

### 2.9 Surface spreading assay

TSB plates containing 0.24% (wt/vol) agar were prepared uniformly in the presence or absence of Nifuratel. Then, the plates were dried for 30 min at room temperature, and equilibrated at 37°C for 1h. *S. aureus* overnight cultures were adjusted to OD_630_ = 0.5, and 2 µL droplets were inoculated centrally onto the agar surface. Plates were incubated statically at 37°C for 24 h and the spreading zone was recorded with a camera (Wu et al., 2024).

### 2.10 Auto-aggregation

Overnight cultured *S. aureus* was collected by centrifugation at 16,000 × g for 2 min, and re-suspended in 3 mL 1× PBS, in the presence or absence of Nifuratel. The suspension was incubated statically at 37°C for 24h and the turbidity was monitored by measuring the OD630nm (Yu et al., 2021).

### 2.11 Resistance detection by serial passage

Log-phased *S. aureus* cultures were diluted and the initial MIC was determined by broth microdilution assay as described above. After incubation at 37°C for 24 h, the MIC was recorded, and the bacterial suspension in the 1/2× MIC wells were 1:1000 diluted with fresh MH broth subsequent passages. The MIC was detected daily for a consecutive 24 passages. CIP was used as a positive control (Song et al., 2021).

### 2.12 Resistance detection by one step development

Prepare agar (MH broth, 17 g/L agarose) with indicated concentrations of Nifuratel or CIP (positive control). Then, 100 μL of *S. aureus* suspension with 10^8^ CFU/mL of bacterial cells was inoculated on the surface of the agar. After incubated at 37°C for 48 h, the CFUs on the plates were counted (Smith et al., 2018).

### 2.13 Murine abscess model

Specific pathogen-free outbred female ICR mice, aged six to seven weeks, were anesthetized using 1% sodium pentobarbital at a dosage of 50 mg/kg. Their back hair was removed by shaving. Fifty microliters *S. aureus* ATCC 43300 bacterial suspension containing 1× 10^8^ CFU/mL was administered by subcutaneous injection to establish infection. The mice were randomly assigned to two groups with six animals each: one group received a dose of DMSO as vehicle control while the other was treated with 30 mg/kg Nifuratel. Both groups received their respective subcutaneous treatments 1h after infection. Twenty-four hours post-infection, the mice were humanely euthanized and the resulting abscesses were surgically excised. The collected abscesses were then either homogenized for quantifying bacterial load or fixed in 4% paraformaldehyde solution for subsequent histological and immunohistochemical analysis (Pletzer et al., 2018).

### 2.14 Wound infection model

Female ICR mice aged 6–8 weeks were anesthetized intraperitoneally with 1% sodium pentobarbital, after which the dorsal skin was shaved and disinfected with 75% ethanol. A full-thickness excisional wound 6 mm in diameter was created, penetrating both the epidermis and dermis. The wound was then inoculated 50 μL of MRSA ATCC 43300 at the concentration of 1×108 CFU/mL. After 1h post infection, topical treatment was administered by applying 2% (wt/vol) of the test compound directly to the wound site once every 24 h for a total of 7 days. At the experimental endpoint, wound tissues were excised for analysis: bacterial burden was quantified by homogenizing tissue in PBS and performing serial dilution plating for CFU counts. Histopathological evaluation was conducted on 4% paraformaldehyde-fixed, paraffin-embedded sections stained with H&E staining (Rehberg et al., 2020).

### 2.15 *In vivo* toxicity

Mice were randomly assigned to two groups (n=6 per group) and received an intraperitoneal injection of either the vehicle control (5% Cremophor EL combined with 5% ethanol) or 30 mg/kg Nifuratel. At 24h post-injection, blood was collected for the quantification of hematological parameters and organic biomarkers. Concurrently, major organs, including the heart, liver, spleen, lungs, and kidneys, were harvested and fixed in 4% paraformaldehyde solution for subsequent histological examination by H&E staining (Wu et al., 2023).

### 2.16 Statistical analysis

All experiments were performed with three independent replicates. Statistical analyses were carried out using GraphPad Prism 9.0 software, employing the Student’s *t*-test for comparisons between two groups and one-way ANOVA for comparisons among multiple groups. A *P*-value of less than 0.05 was considered statistically significant.

Additional details regarding the materials and methods are described in the **Supplementary Information**.

## 3 Results

### 3.1 *In vitro* bactericidal activity of Nifuratel against MRSA

**Figure 1:**
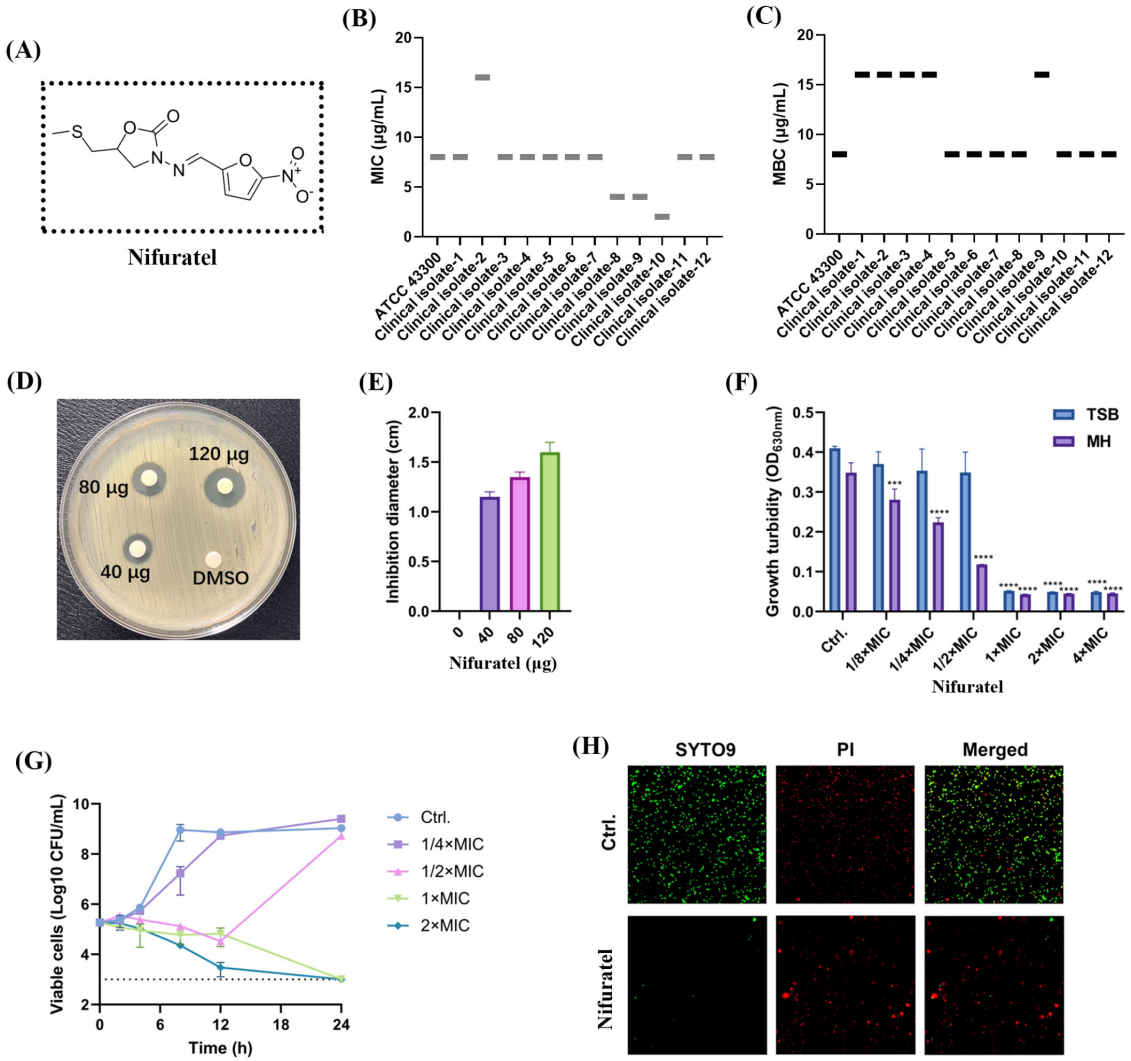
Bactericidal activity of Nifuratel against MRSA. **(A)** 2D chemical structure of Nifuratel. **(B)** MIC values distribution of Nifuratel against MRSA type strain ATCC 43300 and clinical isolates. **(C)** MBC values distribution of Nifuratel against these MRSA strains. **(D)** Growth inhibitory effects against ATCC 43300 determined by K-B test. **(E)** Quantification of the diameters of the inhibition zones. **(F)** Concentration-dependent growth inhibitory effects against ATCC 43300 by Nifuratel in TSB or MH broth. **(G)** Time-killing curve of Nifuratel against ATCC 43300. Dotted line: limit of detection. **(H)** Bacterial cell viability detection by STYO9/PI staining. The log-phased cells of ATCC 43300 were treated with 2× MIC of Nifuratel for 2h. ***P < 0.001. ****P < 0.0001.

Nifuratel, 5-[(Methylthio)methyl]-3-[[(5-nitro-2-furyl)methylene]amino]-2-oxazolidinone (**Figure 1A**), exhibited effective bactericidal activity against *S. aureus*. The MIC and MBC values against MSSA and MRSA type strains and clinical isolates were consistently 2-8 μg/mL and 8–16 μg/mL, respectively (**Figures 1B, C**). Notably, Nifuratel also demonstrated moderate efficacy against *E. faecalis* ATCC 29212 with MIC of 16 μg/mL. In K-B disk diffusion assay, Nifuratel showed concentration-dependent zones of inhibition against MRSA ATCC 43300 (**Figures 1D, E**) as well as other type strain and clinical isolate (**Supplementary Figures S1A, 1B**). And enhanced antimicrobial susceptibility of Nifuratel at sub-MICs was observed in MH broth when compared with TSB (**Figure 1F**), which suggested cation composition in broth may influences the antimicrobial efficacy by Nifuratel. By time-killing assay, Nifuratel exhibited concentration- and time-dependent bactericidal activity against *S. aureus* by both type strains and clinical isolate (**Figure 1G** and **Supplementary Figure S2**). For example, Nifuratel treatment led to CFU reduction at the concentration of 1–2× MIC against ATCC 43300 with no detectable colonies at the time point of 24h (**Figure 1G**). In consistence, SYTO9/PI viability staining visualized bacterial damage with enhanced PI uptake (red fluorescence) after 1h exposure to 1× MIC of Nifuratel (**Figure 1H**).

**Figure 2:**
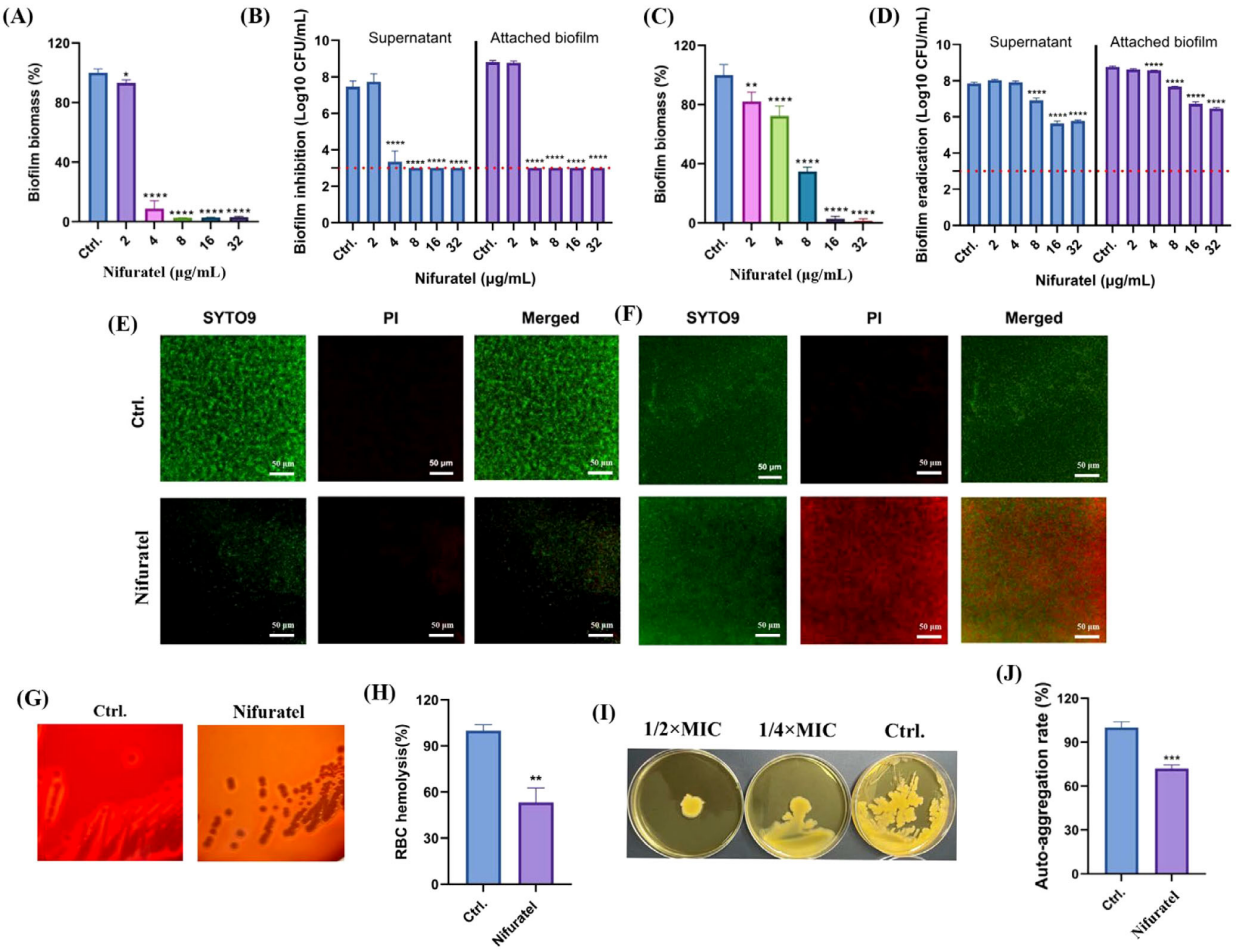
Antibiofilm and anti-virulence activity by Nifuratel. **(A, B)** Biofilm inhibitory activity against ATCC 43300 determination by crystal violet staining **(A)** and CFU counting assay **(B)**, respectively. Dotted line indicates limit of detection. **(C, D)** Pre-formed biofilm eradicating activity against ATCC 43300 determination by crystal violet staining **(C)** and CFU counting assay **(D)**, respectively. Dotted line indicates limit of detection. **(E, F)** Biofilm inhibition **(E)** and eradication **(F)** activities detection by SYTO9/PI staining. The concentrations of Nifuratel used for the biofilm inhibitory and eradicating assays were 4 and 8 μg/mL, respectively. **(G)** Hemolytic activity of MRSA on the sheep blood agars in the presence or absence of 4 μg Nifuratel. SA-11 was selected for this assay due to its strong hemolytic activity. **(H)** Hemolytic activity quantitative of Nifuratel at the concentration of 1/2× MIC. **(I)** Surface spreading inhibition by sub-MICs of Nifuratel. **(J)** Auto-aggregation of ATCC 43300 in the presence or absence of 1/2× MIC Nifuratel. *P < 0.05. **P < 0.01. ***P < 0.001. ****P < 0.0001.

Nifuratel effectively inhibited *S. aureus* biofilm formation and virulence factor production at sub-MICs. Crystal violet staining demonstrated that 4 μg/ml of Nifuratel significantly inhibited biofilm formation (**Figure 2A**). In accordance, Nifuratel reduced the number of viable cells in both the supernatant and the adherent biofilms (**Figure 2B**). Although crystal violet staining indicated that Nifuratel was ineffective against preformed biofilms (**Figure 2C**), it significantly decreased the CFUs in both the supernatant and biofilm-associated cells at concentrations equal to or greater than 8 μg/mL (**Figure 2D**). These results suggest that the antibiofilm effects of Nifuratel are likely attributable to growth inhibition rather than suppression of extracellular matrix production. Consistently, fluorescence imaging using SYTO9/PI revealed that Nifuratel inhibited the biofilm development with decreased overall fluorescence intensity (**Figure 2E**). Despite unchanged total biomass, the mature biofilms treated with Nifuratel exhibited increased PI signals indicating enhanced cells damage in biofilms (**Figure 2F**). Furthermore, sub-MICs of Nifuratel inhibited *S. aureus* key virulence phenotypes including hemolytic activity (**Figures 2G, H**), agar surface diffusion capacity (**Figure 2H**), and Auto-aggregation (**Figure 2I**). These results collectively demonstrate the effectively antimicrobial activity of Nifuratel against *S. aureus* and its virulence factors.

### 3.2 Low resistance development potential of Nifuratel

**Figure 3:**
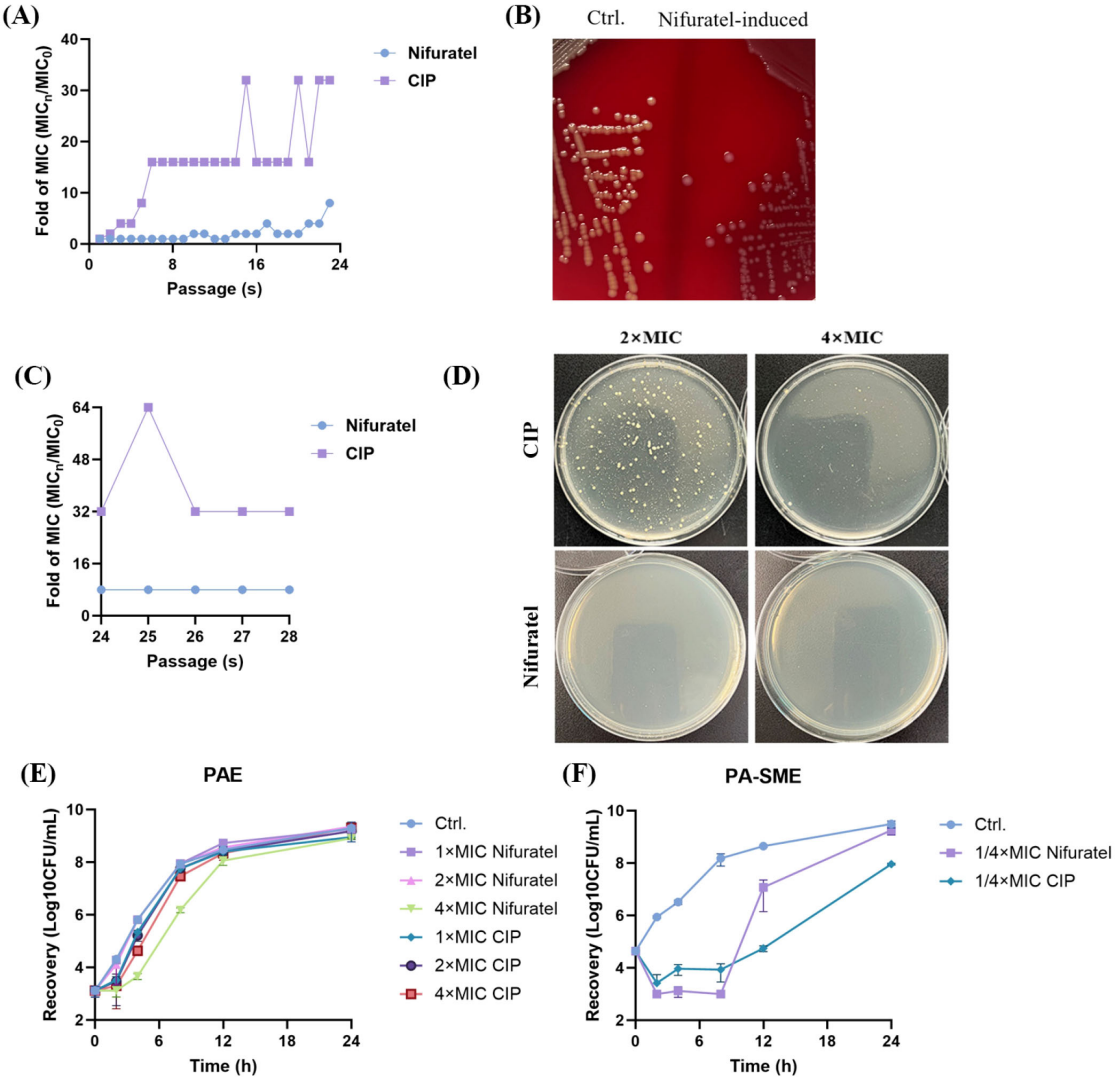
Resistance development potential and PAE/PA-SME of Nifuratel against MRSA ATCC 43300. **(A)** Resistance inducing in the presence of sub-MIC (1/2×MIC) of Nifuratel for consecutive 24 passages. **(B)** Hemolytic activity of the parental strain and its Nifuratel-induced final passage. **(C)** Resistance reversion in CIP-/Nifuratel-induced final passage of *S. aureus*. **(D)** Single-step resistance development by 2-4× MIC of CIP or Nifuratel. **(E)** PAE of CIP and Nifuratel. **(F)** PA-SME of CIP and Nifuratel.

Serial passage experiments revealed obvious differences in resistance development between Nifuratel and CIP. After 23 passages under sub-MIC, CIP induced a 32-fold increase in MIC against *S. aureus* ATCC 43300 whereas Nifuratel caused only a 2-fold MIC elevation (**Figure 3A**). The low resistance development probability by Nifuratel was also observed by ATCC 25923 and SA-11 (**Supplementary Figure S4**). Notably, the Nifuratel-exposed ATCC 43300 from the final passage exhibited reduced Staphyloxanthin production (**Figure 3B**), indicating suppression of virulence factor expression. After five passages in drug-free medium, the MIC values remained unchanged (**Figure 3C**), suggesting that Nifuratel/CIP-induced adaptations may involve genetic mutations rather than phenotypic changes. Furthermore, in single-step resistance selection assay, CIP generated resistant mutants at the concentration of 2–4× MIC, whereas Nifuratel produced no detectable resistant colonies (**Figure 3D**).

The postantibiotic effect (PAE) describes the delayed regrowth of bacteria following short-term antibiotic exposure, mainly caused by persistent cellular damage, such as disruptions in protein synthesis or DNA replication, that takes time to repair (Spangler et al., 1997). In comparison, the postantibiotic sub-MIC effect (PA-SME) prolongs this suppression by introducing sub-inhibitory antibiotic levels after the initial exposure. This further impedes bacterial recovery by continuously disrupting metabolic processes. While both effects enable longer dosing intervals, PA-SME specifically emphasizes how sustained sub-inhibitory drug concentrations can lead to more prolonged growth inhibition than PAE alone (Pankuch and Appelbaum, 2009). In our study, the PAE of Nifuratel was similar as CIP (**Figure 3E**), while its PA-SME surpassed CIP within 8h (**Figure 3F**). This indicates that the cellular damage inflicted by Nifuratel is comparable to that of a fluoroquinolone, but its unique property lies in its enhanced ability to suppress bacterial regrowth at sub-inhibitory concentrations. These data establish Nifuratel as an antimicrobial agent with negligible resistance development capacity and prolonged suppressor effects on bacterial regrowth.

### 3.3 Model of action

**Figure 4:**
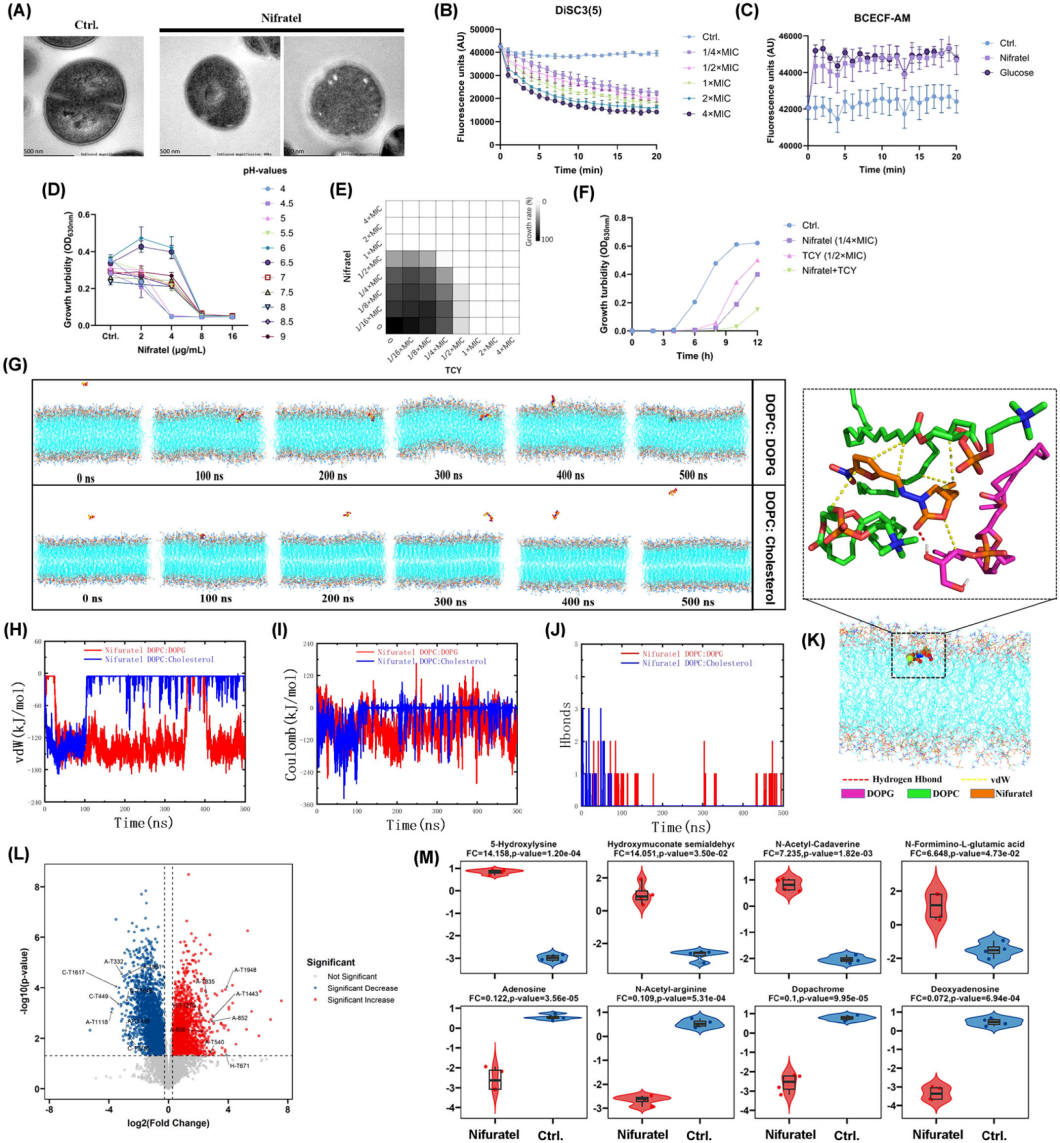
PMF disruption by Nifuratel via interacting with cell membrane phospholipids. **(A)** TEM observation of MRSA ATCC 43300 after treated with 5× MIC of Nifuratel for 1h. **(B)** Transmembrane potential monitoring by DiSC3(5) probe. **(C)** Transmembrane proton gradient monitoring by BCECF-AM probe. **(D)** Growth inhibition activity of Nifuratel in the MH broth with varied pH values. **(E)** Partial synergistic antimicrobial effects between Nifuratel and TCY assessed by checkerboard dilution assay. **(F)** Growth inhibition assay by sub-MICs of Nifuratel/TCY alone or in combination. **(G)** Representative snapshot from the MD simulation of the membrane system. Nifuratel was shown in VDW mode, and the membranes were shown in lines. **(H)** Van der Waals (vdW) interaction energy between Nifuratel and the membranes. **(I)** Electrostatic (Coulomb) interaction energy between Nifuratel and the membranes. **(J)** Number of hydrogen bonds formed between Nifuratel and the membranes during the simulation. **(K)** Detailed interactions between Nifuratel and the DOPC/DOPG membrane during the simulation. **(L)** Volcano plot of differentially abundant metabolites. **(M)** Quantitative analysis of partial differentially abundant metabolites. FC, Fold Change.

As shown in **Figure 4A**, TEM revealed that untreated bacteria exhibited intact ultrastructures, whereas those treated with Nifuratel displayed blurred membrane contours, abnormal inward invaginations of the cytoplasmic membrane, and reduced density of both cell wall and membrane, which suggested possible “edema-like” changes in the periplasmic region. To further investigate the underlying mechanism, we observed that Nifuratel showed concentration-dependent reduction by DiSC3(5) fluorescence intensity (**Figure 4B**), indicating disruption of transmembrane potential-a main component of PMF. Similarly, using the BCECF-AM probe, we found that Nifuratel increased its fluorescence intensity similarly to the positive control glucose, demonstrating interference with the ΔpH, the other key component of PMF (**Figure 4C**). The formation of PMF relies on the dynamic compensation between ΔΨ and ΔpH. By exposing bacteria to varying pH conditions, the relative contribution of ΔΨ and ΔpH to the total PMF shifts, allowing us to infer the mechanism of action of Nifuratel based on changes in antibacterial efficacy. As shown in **Figure 4D**, the growth ability in culture medium was slightly influenced in the presence of varied pH, while obvious growth turbidity was still observed at control groups. As we expected, the antibacterial activity (including the MIC values) of Nifuratel was also notably influenced by external pH alteration (**Figure 4D**). This assay further supporting the PMF-dependent mechanism. Moreover, Nifuratel exhibited a partial synergy (FICI = 0.75) when combined with tetracycline (TCY) (**Figure 4E**) and doxycycline (DOX) (**Supplementary Figure S3**) against *S. aureus*, which was similar with the previous reports for known PMF inhibitors (Ejim et al., 2011). This partial synergism was also corroborated by time-kill curves using sub-MICs of Nifuratel and TCY (**Figure 4F**). Moreover, partial synergistic combinations were also observed between Nifuratel and some conventional antibiotics like daptomycin, ampicillin, and oxacillin, etc (**Supplementary Figure S3**). These effects may arise from mechanisms such as enhanced membrane permeability by the membrane-disrupting agents like daptomycin (Peñalba Arias et al., 2015). Additionally, β-lactam antibiotics can inhibit cell wall synthesis, compromising bacterial integrity and potentiating the activity of other drugs by improving access to targets (Dilworth et al., 2014). Collectively, these findings demonstrate that Nifuratel targeted PMF with boths bacterial transmembrane potential and ΔpH.

MD simulations revealed distinct binding behaviors of Nifuratel toward two types of mixed lipid membranes. As shown in **Figures 4G**, Nifuratel stably bound to and inserted into the DOPC: DOPG bilayer during the simulations, while it rapidly dissociated from the DOPC: Cholesterol membrane after 105 ns. Quantitative analysis indicated that the contact surface area (CSA) (**Supplementary Figure S5A**) and number of atomic contacts (**Supplementary Figure S5C**) increased rapidly after 25 ns and stabilized around 3.5 nm² and 250, respectively, for the DOPC: DOPG system. Although a brief dissociation occurred between 350–400 ns, Nifuratel spontaneously re-embedded into the membrane. In contrast, interaction with the DOPC: Cholesterol membrane was transient and unstable (**Supplementary Figure S5B, C**). As shown in **Figures 4H–K**, Van der Waals (**Figure 4H**) and electrostatic interactions (**Figure 4I**) were the main driving forces, with hydrogen bonding (**Figure 4J**) playing a minor role. Distance analysis corroborated deep and stable embedding of Nifuratel into the DOPC: DOPG bilayer, unlike its temporary binding to the cholesterol-containing membrane (**Supplementary Figure S6**).

By metabolomic analysis, Nifuratel treatment could induce extensive significant alterations in the metabolite profile of *S. aureus* (**Figure 4L** and **Supplementary Table S1**). Key differentially abundant metabolites are summarized in **Figure 4M**. Upregulated metabolites included 5-Hydroxylysine, Hydroxymuconate semialdehyde, N-Acetyl-Cadaverine, et al, while downregulated metabolites consisted of Cyclohexane-1-carboxylic acid, Deoxyguanosine, Acetyl-Glycine, et al. The pronounced accumulation of polyamine derivatives (e.g., N-Acetyl-Cadaverine) coupled with a sharp decline in nucleotide pools (e.g., Adenosine and Deoxyadenosine) points to a profound disruption of cellular energy and biosynthetic homeostasis. These coordinated metabolic shifts are consistent with the collapse of the proton motive force (PMF), which critically governs bacterial energetics and transmembrane transport (Yang et al., 2023). Notably, the concurrent pattern of nucleotide depletion alongside polyamine buildup delineates a metabolic stress signature that may represent a previously uncharacterized bacterial adaptive response to PMF impairment.

### 3.4 Effective antimicrobial effects of Nifuratel *in vivo*

**Figure 5:**
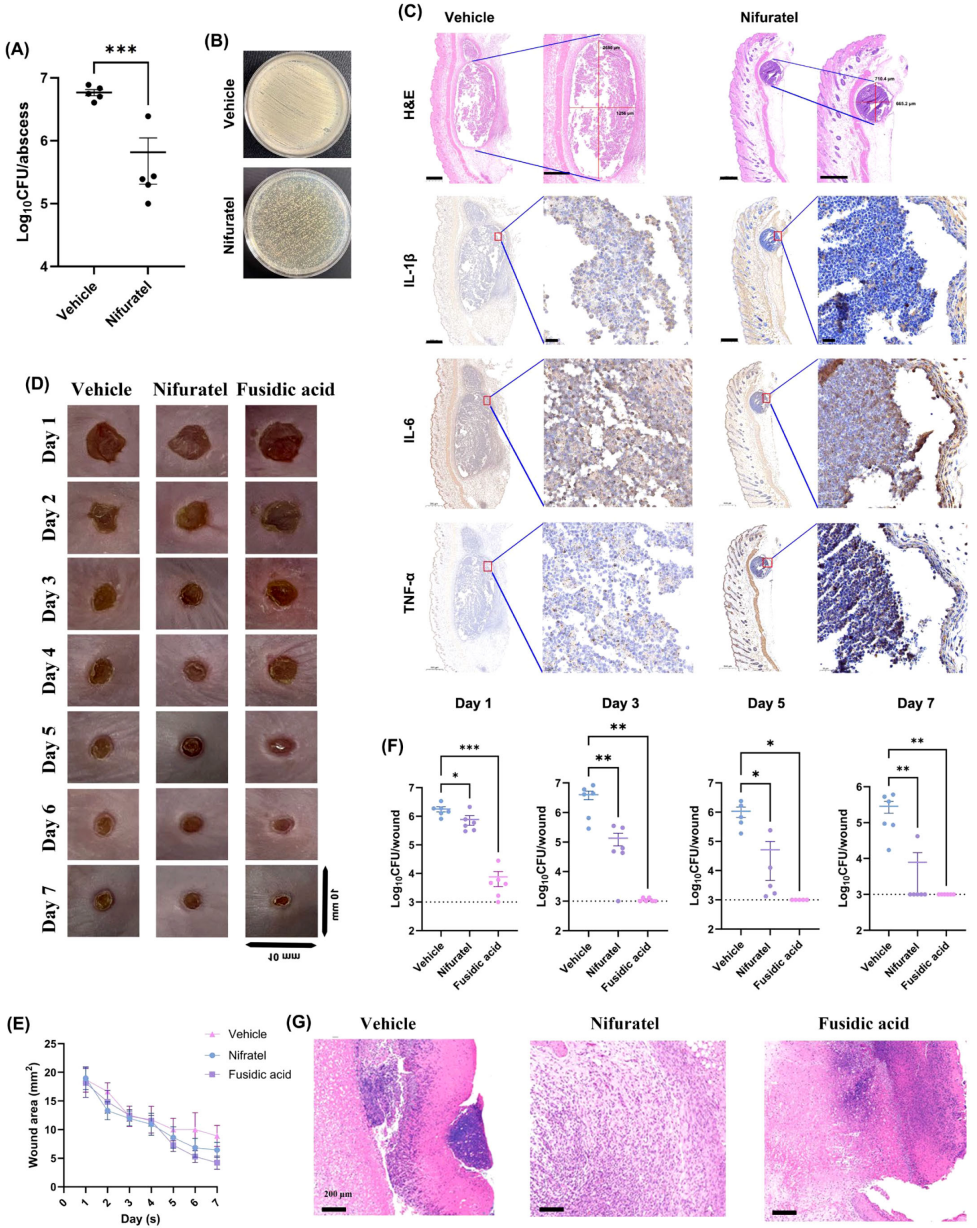
*In vivo* antimicrobial efficacy of Nifuratel in abscess and wound infection models. **(A)** Viable bacterial cells quantification in abscesses. **(B)** Representative images of the viable cells quantification. **(C)** Pathological analysis (including H&E staining and immunohistochemistry) of the abscesses in the treated and untreated groups. **(D)** Representative images of the infected wounds in varied treatment groups during 7 days. **(E)** Wound area quantification at different time points. **(F)** Viable bacterial cells quantification in the wounds at different time points. **(G)** H&E staining of the infected wounds in different treatment groups at day 7. *P < 0.05. **P < 0.01. ***P < 0.001.

To evaluate the antibacterial efficacy of Nifuratel *in vivo*, we employed both abscess and wound infection models. In the abscess model, Nifuratel treatment significantly reduced the viable bacterial load compared to the vehicle group (**Figure 5A**), which was in accordance with the representative images of the abscesses (**Figure 5B**). Histological examination via H&E staining revealed a substantial reduction in both abscess size and total inflammatory infiltration in Nifuratel-treated mice (**Figure 5C**). Additionally, immunohistochemical analysis showed markedly reduced overall expression of cytokines IL-1β, IL-6, and TNF-α after treated with Nifuratel (**Figure 5C**). Similarly, in the wound infection model, a 7-day period observation demonstrated obvious wound closure in the groups treated with Nifuratel or fusidic acid (positive control), in contrast to the vehicle group (**Figures 5D, E**). Quantification of viable bacterial cells in wounds on day 1, 3, 5 and 7 indicated that Nifuratel exhibited time-dependent antibacterial activity, reaching efficacy comparable to fusidic acid by day 7 (**Figure 5F**). Meanwhile, H&E staining of the wounds displayed obvious reduced inflammatory cell infiltration in both Nifuratel and fusidic acid treated groups relative to the Vehicle group (**Figure 5G**). Together, these findings demonstrate that Nifuratel robustly diminishes bacterial loads and alleviates infection-associated inflammation *in vivo*, highlighting its therapeutic potential for treating *S. aureus* skin and soft tissue infections.

### 3.5 Favorable biocompatibility by Nifuratel

**Figure 6:**
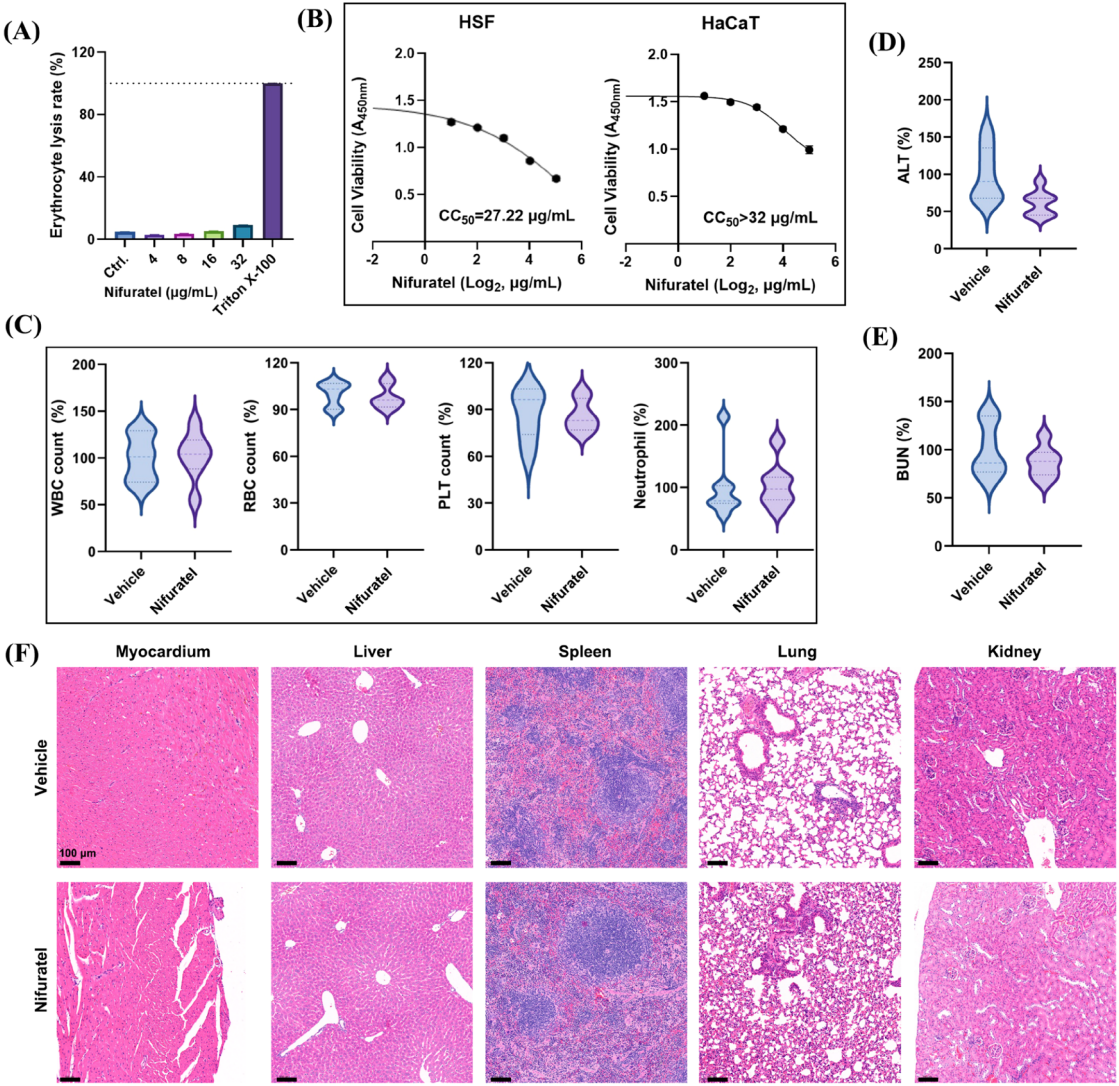
*In vitro* and *in vivo* toxicity assessment. **(A)** Human RBC hemolysis activity of Nifuratel. Triton X-100 (0.1%) was used as a positive control. **(B)** Cytotoxicity determination of Nifuratel against skin epithelial cell lines by CCK-8 kit. CC_50_: half cytotoxic concentration. **(C)** Blood routine parameters quantification. The mice were treated with 30 mg/kg Nifuratel or equal amount of ethanol+ Cremophor EL by intraperitoneal injection. No statistical significance was observed between the vehicle and Nifuratel-treated group. **(D, E)** Quantification of serum ALT (a biomarker for liver function) **(D)** and BUN (a biomarker for renal function) **(E)**, respectively. The mice were treated as described above. No statistical significance was observed between the vehicle and Nifuratel-treated group. **(F)** H&E staining of main organs after treatment with 30 mg/kg Nifuratel or equal amount of ethanol+ Cremophor EL by intraperitoneal injection.

To assess the biosafety of Nifuratel, both *in vitro* and *in vivo* toxicity studies were conducted. As shown in **Figure 6A**, Nifuratel exhibited extremely low hemolytic activity even at concentrations up to 32 μg/mL. Nifuratel also demonstrated low cytotoxicity toward human keratinocyte (HaCaT) and human skin fibroblast (HSF) cell lines with the CC_50_ values of 27.22 μg/mL and > 32 μg/mL, respectively (**Figure 6B**). For *in vivo* toxicity evaluation, mice treated intraperitoneally with 30 mg/kg Nifuratel showed no remarkable changes in routine blood parameters (**Figure 6C**) or specific biomarkers of liver (**Figure 6D**) and kidney function (**Figure 6E**). Consistent with these observations, histopathological examination via H&E staining revealed no apparent pathological alterations in major organs (**Figure 6F**). Collectively, these results indicate that Nifuratel, repurposed as an antimicrobial agent, possesses a high safety profile *in vivo*, supporting its potential for therapeutic application.

## 4 Discussion

This study provides a comprehensive evaluation of the antibacterial activity, mechanism, and safety profile of Nifuratel against *S. aureus*. Our study firstly demonstrated that Nifuratel exerts antibacterial effects against *S. aureus* by disrupting the PMF, while concurrently suppressing bacterial virulence production, and attenuating the evolution of resistance. With notable efficacy and favorable biocompatibility *in vivo*, Nifuratel represents an optimal candidate compound for the treatment of drug-resistant bacterial infections.

Targeting the PMF represents an emerging antibacterial strategy with broad implications. Unlike many conventional antibiotics that inhibit specific enzymes or synthetic pathways, PMF disruption leads to rapid collapse of bioenergetic homeostasis and membrane aberrant (Farha et al., 2013). Interestingly, Nifuratel could simultaneously impair both ΔΨ and ΔpH components of the PMF. This bifunctional action aligns with emerging PMF modulation paradigms while offering distinct advantages in overcoming efflux-mediated resistance through comprehensive energy depletion. In addition, although significant resistance to Nifuratel was not rapidly observed, any subtle shift could be mediated by genetic factors. Therefore, future studies analyzing potential genetic changes will be valuable for fully delineating its antimicrobial mechanism (**Figure 3A**). Recently, many PMF inhibitors against *S. aureus* were reported. For example, the quaternary ammonium compound berberine exhibits potent anti-Staphylococcal activity by increasing bacterial cell membrane permeability and disrupt PMF (Dilworth et al., 2014); Small molecules C218–0546 and its analogue STK848198 disrupt *S. aureus* ATP utilization by targeting the ΔΨ component of PMF (Zhao et al., 2023). By probes tracing and MD simulations, Seo et al (Seo et al., 2024). identified a novel indole-quinoline hybrid molecule, which was found to be effective against *S. aureus* by PMF inhibition. However, Nifuratel distinguishes itself through its additional anti-virulence effects and markedly lower cytotoxicity, which may offer therapeutic advantages over existing PMF-targeting agents.

Nifuratel effective inhibits the virulence, including biofilm formation, surface spreading, hemolytic activity and auto-aggregation, production of *S. aureus* at sub-MICs. These virulence factors are regulated by quorum sensing systems, with the accessory gene regulator (*agr*) and SaeRS systems being among the most extensively studied. The *agr* system facilitates cell-to-cell communication via autoinducing peptides, and its activation primarily leads to the expression of exo-toxins and exo-enzymes. In contrast, the SaeRS system regulates the production of a broader range of exo-proteins, including many key virulence factors (Pengfei et al., 2025). Thus, the anti-virulence activity of Nifuratel at sub-MICs could be mediated through the inhibition of the Agr or SaeRS systems. Similar as our study, Jiang et al (Jiang et al., 2023). reported that the repurposed anti-inflammatory drug Fenoprofen exerts its therapeutic effect by targeting the SaeR regulatory protein in *S. aureus*. This inhibition significantly attenuates the expression of a suite of key bacterial virulence factors without exerting direct bactericidal pressure, which highlights the promising therapeutic potential of Fenoprofen to treat *S. aureus* biofilm-related infections by specifically targeting its virulence regulatory machinery. The work by Wu et al (Wu et al., 2024). reveals that the mitochondrial-targeted antioxidant visomitin inhibits *S. aureus* virulence at sub-MICs by targeting the agr system. This finding indicates the potential of using visomitin in combination with conventional antibiotics as a novel anti-virulence strategy. Collectively, the potential anti-virulence production activity of Nifuratel alone or in combination with antibiotics represents a promising therapeutic strategy to combat MRSA-related infections.

Although, Nifuratel interacts with the bacterial cell membrane and targets the PMF, its direct membrane-disrupting probability is low. As revealed by MD simulations, the primary interactions between Nifuratel and the bacterial cell membrane are van der Waals forces and hydrophobic interactions (**Figures 3H–K**). In contrast to known membrane disruptors such as L007-0069 (Jenul and Horswill, 2019) and tafenoquine (Pengfei et al., 2022), Nifuratel forms significantly fewer hydrogen bonds with membrane components. This reduced capacity for hydrogen bonding likely underlies its weaker interaction with lipid molecules in the membrane, suggesting a distinct mode of action compared to other disruptors. The observation that Nifuratel exhibits time-dependent bactericidal activity (**Figure 1G**) with a low potential for direct membrane lysis suggests that any associated inflammatory response *in vivo* caused by rapid bacterial lysis would be minimal. The remarkably low *in vivo* toxicity of Nifuratel can be attributed to its unique mechanism of action, which relies on the distinct mechanisms of PMF generation in bacterial membranes compared to human mitochondria. This ensures its selective toxicity against bacterial cells while sparing host mammalian cells (Yang et al., 2023). By selectively targeting bacteria with minimal human cell toxicity, this compound could achieve a breakthrough in therapeutic window compared to nephrotoxic agents like telavancin (Cavanaugh et al., 2019).

A notable characteristic of Nifuratel is its selective activity against Gram-positive bacteria including *S. aureus*, with limited efficacy against Gram-negative pathogens. This may be attributed to the inability of Nifuratel to traverse the outer membrane of Gram-negative bacteria, which serves as a permeability barrier (Hancock and Bell, 1988). Future studies should assess potential impacts of plasma protein binding and pharmacokinetic optimization to enhance its systemic applicability. Despite these limitations, the high antibacterial activities and low resistance propensity position Nifuratel as a promising therapeutic candidate against skin and soft tissue infections.

## Data Availability

The original contributions presented in the study are included in the article/**Supplementary Material**. Further inquiries can be directed to the corresponding author.
