# Supplementary material for: Drug repurposing of Nifuratel against methicillin-resistant Staphylococcus aureus through proton motive force disruption
Source: Front Cell Infect Microbiol. 2026 Jan 9;15:1738031. doi: 10.3389/fcimb.2025.1738031 (PMC12827513; doi:10.3389/fcimb.2025.1738031)
Supplement: Supplementary file 2 [file Table2.docx]

Supplementary Material

**1. Supplementary Materials and Methods**

1.1. Post-Antibiotic Effect (PAE)

Log-phased *S. aureus* was adjusted to ~1×10^6^ CFU/mL with 1-4× MIC of Nifuratel. After incubation at 37°C 200 rpm for 1h, the antimicrobial was removed by 1:1000 dilution with fresh TSB. Then, the bacterial growth was monitored by viable counts (CFU/mL) at intervals of 0, 2, 4, 6, 12, and 24 h, respectively[1].

1.2. Post-Antibiotic Sub-MIC Effect (PA-SME)

Log-phased *S. aureus* was adjusted to ~1×10^6^ CFU/mL in the presence or absence of 4× MIC Nifuratel for 1h. Then, the suspension was 1:1,000 diluted with fresh TSB containing 1/4× MIC of Nifuratel. CIP was used as a control. The bacterial growth was monitored by viable counts (CFU/mL) at intervals of 0, 2, 4, 6, 12, and 24 h, respectively[1].

1.3. Haemolysis activity quantification

Log-phased *S. aureus* was incubated with 1/2×MIC of Nifuratel at 37 ℃ 200 rpm for 24h. Then, 25 μL of commercialized human RBCs (Hemo Pharmaceutical and Biological Co.) were added with the the Nifuratel-treated *S. aureus*. After incubated at 37 ℃ for 60 min, the absorbance at 570nm was recorded following centrifugation at 3000×g for 5 min [2].

1.4. Ultrastructure observation by transmission electron microscopy (TEM)

TEM was employed to visualize ultrastructural alterations in bacteria following Nifuratel treatment. Briefly, log-phased S. aureus was diluted to ~1× 10^6^ CFU/mL in the presence or absence of 5×MIC of Nifuratel. After incubation at 37 °C 200 rpm for 1h, the cells were collected and washed with PBS by centrifugation. Then, the samples were fixed in 2.5% glutaraldehyde and 1% osmium tetroxide, dehydrated through an ethanol gradient, embedded in Spurr’s resin, and sectioned (60–80 nm) for imaging[3].

1.5. Membrane potential determination by DiSC3(5) probe

*S. aureus* cells were harvested during mid-exponential growth, washed, and re-suspended in assay buffer (5 mM HEPES, 5 mM glucose, pH 7.0–7.4) supplemented with 100 mM KCl. Cells were added with 2 μM DiSC3(5) and incubated in the dark until fluorescence stabilization. After adding Nifuratel or Melittin (positive control) at indicated concentrations, the fluorescence intensity was monitored at the excitation/emission wavelengths of 622nm/670nm, respectively, using a microplate reader (PerkinElmer EnVision, USA)[4].

1.6. Transmembrane proton gradient (△pH) determination by BCECF-AM probe

Log-phased *S. aureus* cultures were harvested by centrifugation. The pellet was washed with 1× PBS and resuspended in 5 mM HEPES buffer in the presence of 10 μM BCECF-AM. 20 mM glucose was used as a positive control. After incubated at 37°C for 30 min in darkness, the bacterial suspension was washed by PBS to remove excess dye, and indicated concentrations of Nifuratel were added to each well. Fluorescence intensity (excitation 488 nm/emission 535 nm) was monitored at indicated time points using the microplate reader as described above[5].

1.7. Checkerboard dilution assay

Serial 2-fold dilutions of each compound were prepared in MH broth and added into a 96-well microplate, generating orthogonal concentration gradients (one diluted along rows, the other along columns). Then log-phased bacterial suspensions were inoculated into each well to the final concentration of ~5 × 10^5^ CFU/mL. After incubated aerobically at 37°C for 16–18h, the MICs were determined by measuring the OD630nm. And the fractional inhibitory concentration index (FICI) was calculated as: FICI = [MIC_A(combination)_/MIC_A(alone)_] + [MIC_B(combination)_/MIC_B(alone)_]. The interactions outcomes were interpreted as: synergy (FICI ≤ 0.5), partial synergy (0.5 < FICI < 1), addition (FICI= 1) or antagonism (FICI > 4)[6].

1.8. Molecular dynamics (MD) simulation

The MD simulation protocol was initiated by constructing the initial structure of Nifuratel using Chem3D software, which was subsequently optimized at the DFT/B3LYP/6-311G(d) level with Gaussian 16, followed by RESP charge calculation using Multiwfn 3.8. Two mixed lipid bilayers, a DOPC: DOPG (prokaryote, 7:3, 294:126 molecules) and a DOPC: Cholesterol (eukaryote, 7:3, 350:150 molecules) membrane, were built and centrally placed in simulation boxes of dimensions 12.038×12.038×13.353 nm³ and 12.228×12.228×13.353 nm³, respectively, using Gromacs 2023.3. A single nifuratel molecule was positioned above each membrane, after which the systems were solvated with water, neutralized, and brought to a physiological ion concentration of 0.15 mol/L with Na⁺ and Cl⁻ ions, resulting in systems containing 183,250 and 188,145 atoms. The CHARMM36 force field described the lipids and cholesterol, the CGenFF force field was used for nifuratel, and water was modeled with TIP3P. All simulations, performed with Gromacs 2023.3, involved energy minimization via the steepest descent algorithm, followed by 500 ps of NVT and 500 ps of NPT equilibration. Production runs were conducted for 500 ns at 300 K and 1 bar, maintained by the V-rescale thermostat and C-rescale barostat, using a 2 fs timestep, a 12 Å cutoff for van der Waals interactions, and the PME method for electrostatics. Trajectories were saved every 10 ps for analysis with VMD and PyMOL under periodic boundary conditions[6, 7].

1.9. Metabolomics

Log-phased *S. aureus* was treated with 5×MIC of Nifuratel at 37 ℃ 200 rpm for 1h. Then, the bacterial cells were washed with 1× PBS and collected by centrigufation. DMSO was used as a contrl. A pre-chilled methanol/water (4:1, v/v) solution was used to metabolite extraction. Bacterial samples were homogenized on ice in extraction solution with metal beads using a homogenizer (3 cycles of 20 s each). The homogenates were vortexed, incubated at -20 °C for 10 min, and centrifuged (12,000 rpm, 10 min, 4 °C) to obtain supernatant for subsequent chemical isotope labeling. For each metabolite class-amines/phenols, carboxyls, hydroxyls, and carbonyls-aliquots were reconstituted in a specified solvent (MS-grade water or acetonitrile/water) and labeled according to kit protocols. After LC-UV quantification of the amine/phenol sub-metabolome, an equal amount of the 13 C-labeled pool was added to each ^12^C-labeled individual sample for LC (Agilent 1290, USA) linked to Q-TOF Mass Spectrometer (MS, Agilent 6546, USA) analysis, with a quality control sample prepared by mixing the ^12^C- and ^13^C-labeled pools. Data were acquired via the LC-MS and processed using IsoMS Pro 1.4.0[8].

1.10. Human red blood cells (RBC) hemolysis assay

The hemolytic activity of the test compounds was assessed using commercialized human RBCs (Hemo Pharmaceutical and Biological Co.). Briefly, the RBC was washed and re-suspended in 1× PBS to the concentration of 4% (v/v). After incubation with indicated concentrations of Nifuratel at 37°C for 1h, the samples were centrifuged (1,500 × g, 10 min), and the absorbance of the supernatant was measured at 570 nm. In addition, 0.1% Triton X-100 and 1% DMSO were served as the positive and negative controls, respectively[9].

1.11. Cytotoxicity determination by CCK-8 kit

Cells (HaCaT and HSF) were seeded in 96-well plates at a density of 1 × 10⁴ cells/well in complete medium (DMEM supplemented with 10% FBS) and incubated for 24 hours at 37°C under 5% CO₂ to allow for adhesion. Following attachment, the medium was replaced with fresh medium containing serially diluted Nifuratel, with 0.1% DMSO serving as the negative control, and the plates were incubated for another 24 hours. Thereafter, 10 μL of CCK-8 reagent was added to each well, followed by incubation at 37°C with 5% CO₂ for 3 hours Absorbance was subsequently measured at a wavelength of 450 nm using a microplate reader[10].

**References**

1. Pankuch GA, Appelbaum PC (2009) Postantibiotic effect of ceftaroline against gram-positive organisms. Antimicrob Agents Chemother 53(10): 4537-4539

2. Wu R, Wu Y, Wu P, Li H, She P (2024) Bactericidal and anti-quorum sensing activity of repurposing drug Visomitin against *Staphylococcus aureus*. Virulence 15(1): 2415952

3. Zhang S, Qu X, Tang H, Wang Y, Yang H, Yuan W et al (2021) Diclofenac resensitizes methicillin-resistant *Staphylococcus aureus* to beta-lactams and prevents implant infections. Adv Sci (Weinh) 8(13): 2100681

4. Miyazaki H, Midorikawa N, Fujimoto S, Miyoshi N, Yoshida H, Matsumoto T (2017) Antimicrobial effects of lysophosphatidylcholine on methicillin-resistant *Staphylococcus aureus*. Ther Adv Infect Dis 4(4): 89-94

5. Liu Y, Jia Y, Yang K, Li R, Xiao X, Zhu K et al (2020) Metformin restores tetracyclines susceptibility against multidrug resistant bacteria. Adv Sci (Weinh) 7(12): 1902227

6. She P, Yang Y, Li L, Li Y, Liu S, Li Z et al (2023) Repurposing of the antimalarial agent tafenoquine to combat MRSA. mSystems 8(6): e0102623

7. Kim W, Zhu W, Hendricks GL, Van Tyne D, Steele AD, Keohane CE et al (2018) A new class of synthetic retinoid antibiotics effective against bacterial persisters. Nature 556(7699): 103-107

8. Wong EHJ, Ng CG, Goh KL, Vadivelu J, Ho B, Loke MF (2018) Metabolomic analysis of low and high biofilm-forming *Helicobacter pylori* strains. Sci Rep 8(1): 1409

9. Pengfei S, Yifan Y, Linhui L, Yimin L, Dan X, Shaowei G et al (2025) Novel antibiotics against *Staphylococcus aureus* without detectable resistance by targeting proton motive force and FtsH. MedComm (2020) 6(1): e70046

10. Ding X, Yang C, Moreira W, Yuan P, Periaswamy B, de Sessions PF et al (2020) A macromolecule reversing antibiotic resistance phenotype and repurposing drugs as potent antibiotics. Adv Sci (Weinh) 7(17): 2001374


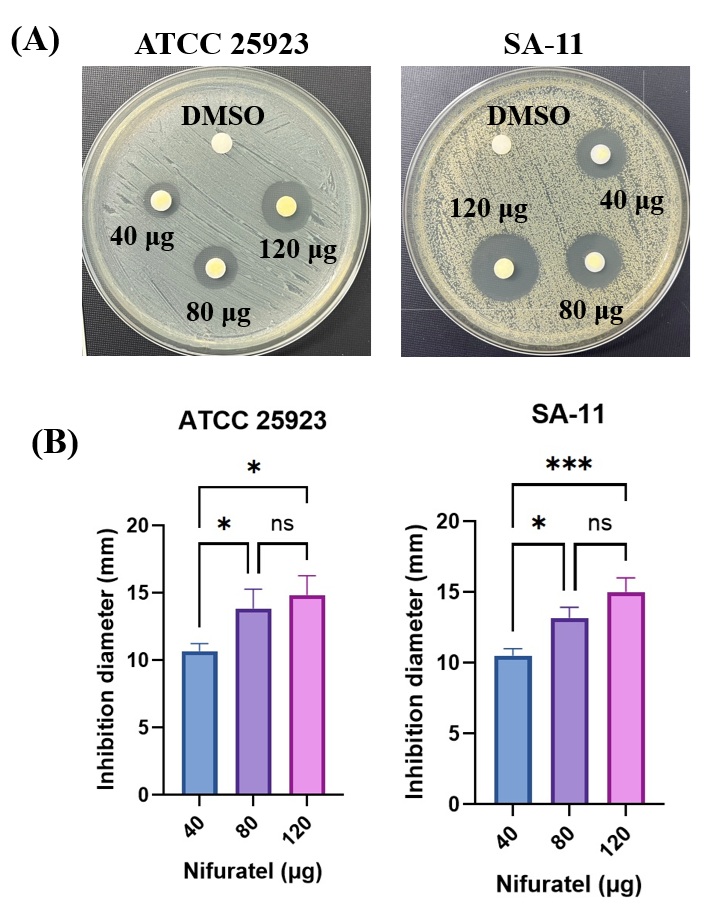


**Supplementary Figure S1.** Dose-dependent growth inhibitory effects of Nifuratel against *S. aureus* ATCC 25923 and SA-11. (A) Antimicrobial activity determination by K-B test. (B) Quantification analysis of the inhibition diameters in the K-B test.


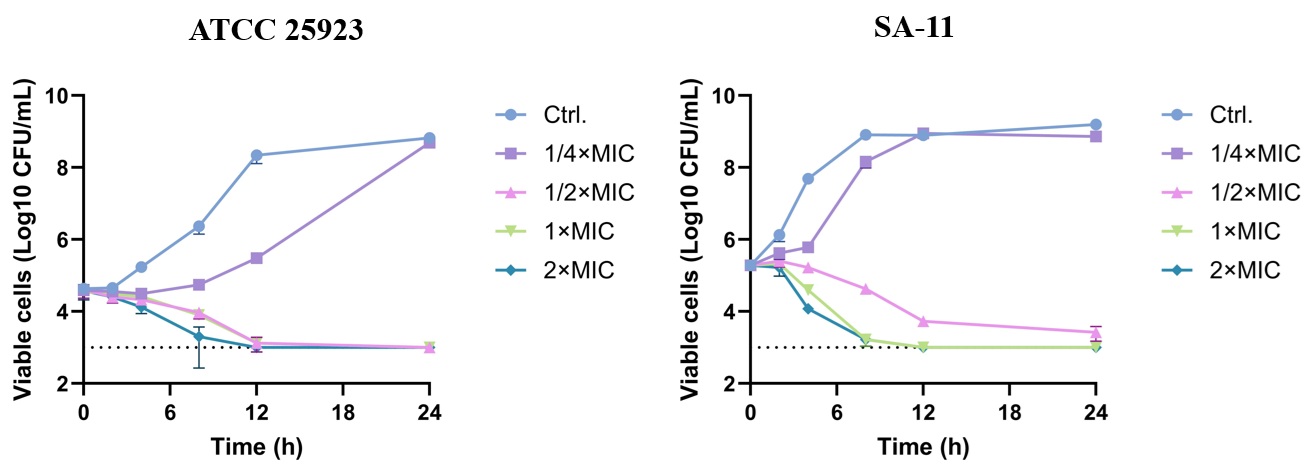


**Supplementary Figure S2.** Time-killing curves of Nifuratel against *S. aureus* ATCC 25923 and SA-11.


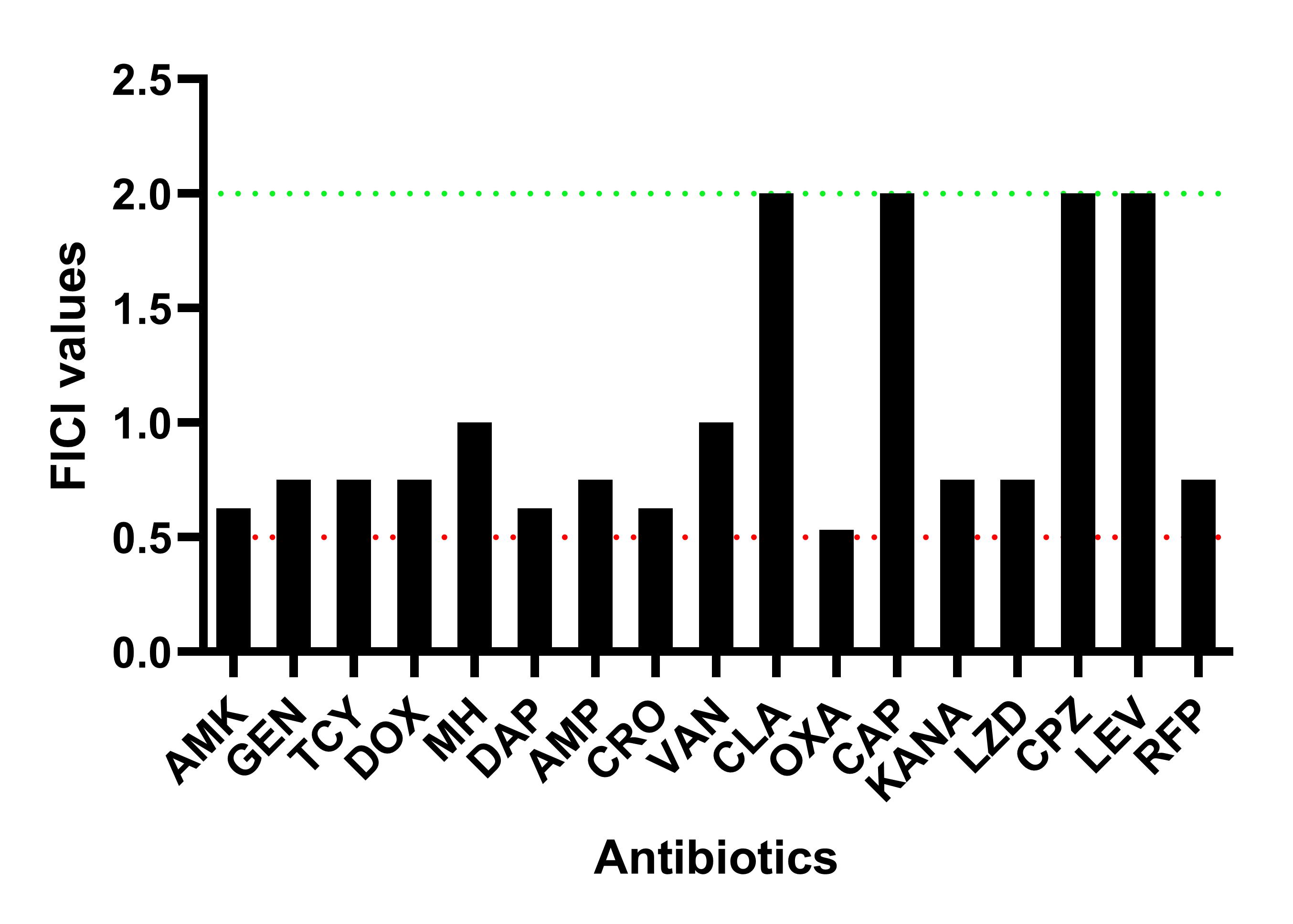


**Supplementary Figure S3.** Drug combinational effects between Nifuratel and conventional antibiotics. AMK: amikacin, GEN: gentamycin, TCY: tetracycline, DOX: doxycycline, MH: minocycline, DAP: daptomycin, AMP: ampicillin, CRO: ceftriaxone, VAN: vancomycin, CLA: clarithromycin, OXA: oxacillin, CAP: chloramphenicol, KANA: kanamycin, LZD: linezolid, CPZ: cefoperazone, LEV: levofloxacin, RFP: rifampicin.


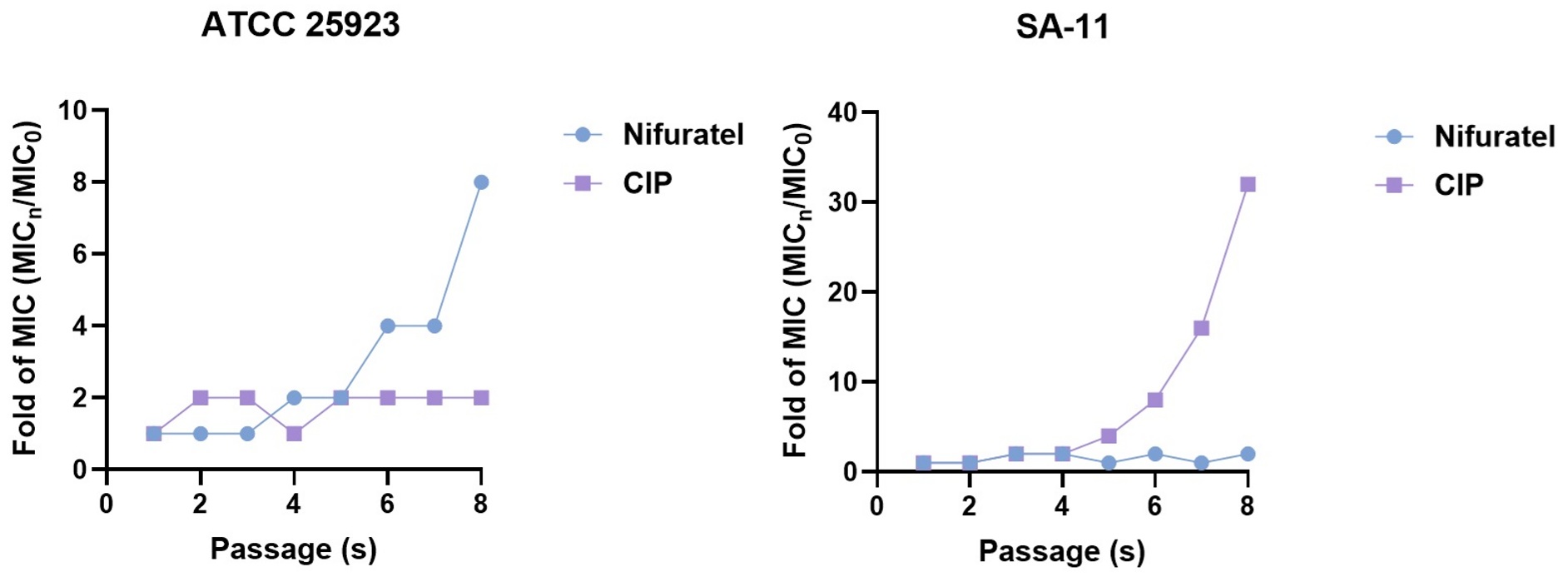


**Supplementary Figure S4.** Resistance inducing ability by sub-MICs of Nifuratel or CIP.


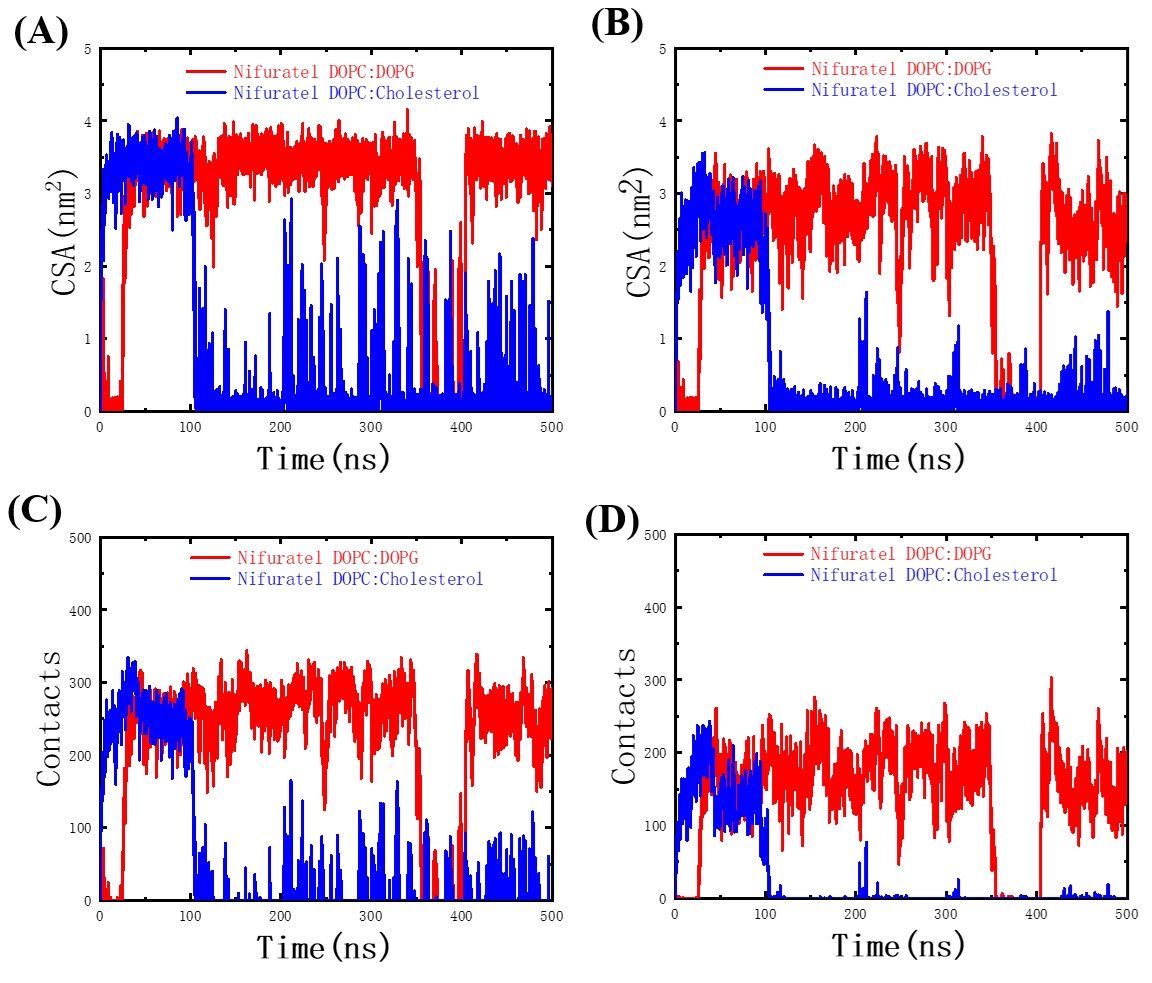


**Supplementary Figure S5.** Analysis of the contact between Nifuratel and cell membranes or its hydrophobic regions during MD simulations. (A) Contact surface area (CSA) between Nifuratel and cell membranes. (B) CSA between Nifuratel and the hydrophobic tails of the membranes. (C) Number of contacting atoms between Nifuratel and the cell membranes. (D) Number of contacting atoms between Nifuratel and the hydrophobic tails of the membranes.


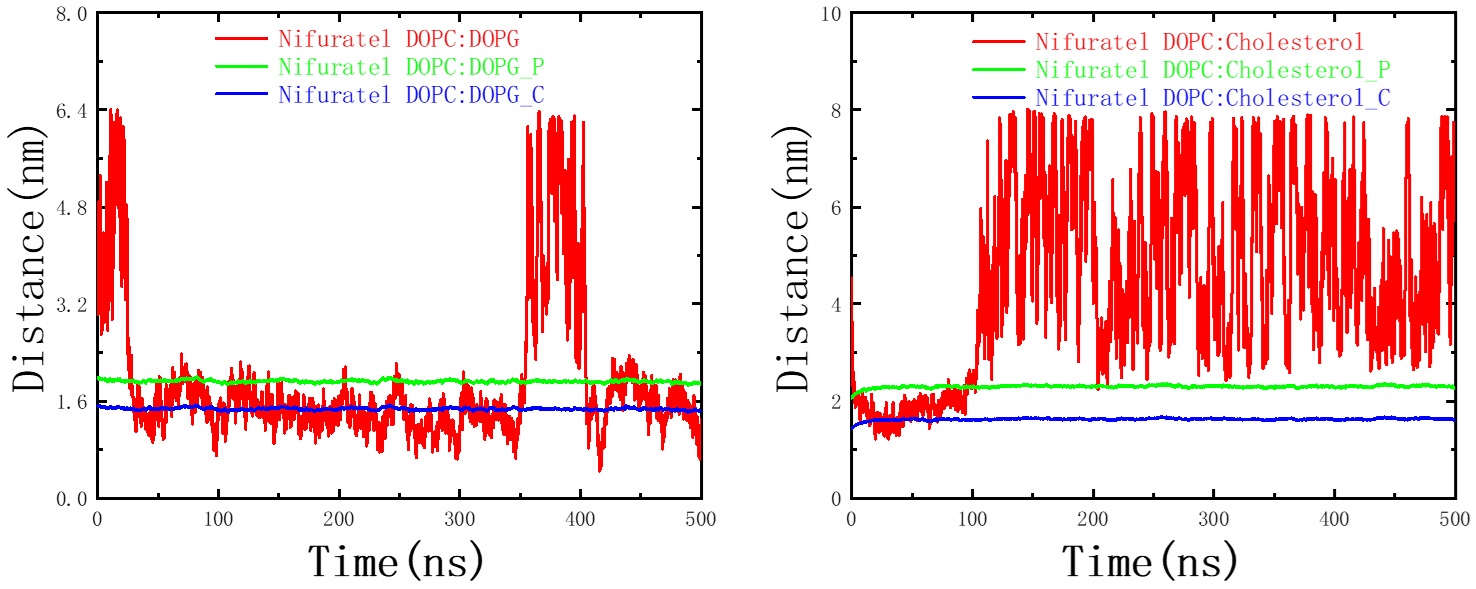


**Supplementary Figure S6.** Analysis of center-of-mass distances during MD simulations. (A) Distance between Nifuratel and the center-of-mass of the DOPC: DOPG membrane. (B) Distance between Nifuratel and the center-of-mass of the DOPC: Cholesterol membrane. ‘P’ and ‘C’ indicated the position of the phosphorus atoms and carbonyl carbon atoms in the phospholipid molecules, respectively.
